# Emotional regulation in alcohol use disorder (AUD)

**DOI:** 10.1192/j.eurpsy.2021.1529

**Published:** 2021-08-13

**Authors:** S. Ferreira, L. Moutinho, J. Teixeira

**Affiliations:** Utra, CHPL, Lisboa, Portugal

**Keywords:** emotions, alcohol use disorders, emotional regulation

## Abstract

**Introduction:**

The process of emotional regulation allows the patient to deal with various situations throughout life, since it includes the ability to create and control emotions, in order to guide action and interaction with others. However, people with alcohol use disorder (AUD) is not always able to give appropriate responses to surronding situations in the face of certain specific emotions.

**Objectives:**

We aimed to evaluate the use of emotional regulation strategies in people with AUD.

**Methods:**

A descriptive and correlational study was conducted. A sociodemographic questionnaire, that included variables to assess aspects related to AUD, and the Emotion Regulation Questionnaire were used.

**Results:**

The sample had 25 participants, mostly male, average age of 46.68 years. 44% were married, and most cases had an withdrawal time larger than 3 months. Regarding emotional regulation strategies, it was found that the participants resort more to cognitive reassessment (M=26.59,SD = 7.54), compared to emotional suppression (M = 15.16, SD = 5.03). Statistically significant differences were found between genders in relation to cognitive reassessment (U = 29.00; p = 0.02). No correlations were found between withdrawal time, treatment time, cognitive reevaluation and emotional suppression.

**Conclusions:**

Results show differences between gender, and the absence of a relationship between educational qualifications in cognitive reassessment, contradicting previous findings on general Portuguese population. This data points to the need to implement intervention programs in this population, taking into account the gender variable, and including the development of the ability to identify and express emotions, as well as of strategies to deal with emotional aspects.

